# Validating the Gender Variance Scale in Italian: Psychometric Properties and Associations with Health and Sociodemographic Factors

**DOI:** 10.3390/healthcare13192438

**Published:** 2025-09-25

**Authors:** Paolo Meneguzzo, David Dal Brun, Elena Tenconi, Marina Bonato, Alberto Scala, Marina Miscioscia, Andrea Garolla, Angela Favaro

**Affiliations:** 1 Department of Neuroscience, University of Padova, 35122 Padova, Italy; 2 Padova Neuroscience Center, University of Padova, 35122 Padova, Italy; 3 Regional Reference Center for Gender Incongruence, Azienda Ospedale-Università di Padova, 35128 Padova, Italy; 4 Department of Developmental Psychology and Socialization, University of Padova, 35122 Padova, Italy; 5 Unit of Andrology and Reproductive Medicine, Department of Medicine, University of Padova, 35122 Padova, Italy

**Keywords:** gender identity, gender variance, transgender individuals, psychometric validation, mental health assessment

## Abstract

**Background:** The Gender Variance Scale (GVS) was developed to assess self-perceived masculinity and femininity across diverse gender identities, including binary and non-binary experiences. To date, no validated Italian version was available. **Methods:** A total of 356 participants (192 transgender and gender-diverse [TGD], 164 cisgender) completed the Italian GVS and the SF-12 Health Survey. Translation and cultural adaptation followed international guidelines. Psychometric evaluation included confirmatory factor analysis (CFA), internal consistency, test–retest reliability (*n* = 63), convergent validity with health-related quality of life, and group comparisons across gender identity categories. **Results:** CFA supported the original two-factor model (CFI = 0.916, TLI = 0.905, RMSEA = 0.076, SRMR = 0.053). Internal consistency was high (α = 0.89). The GVS distinguished between gender identity groups: TGD participants scored higher than cisgender peers, and non-binary individuals reported significantly lower scores than both binary groups. Test–retest reliability was strong (r = 0.87–0.99; ICC = 0.992–0.996). **Conclusions**: The Italian GVS is a valid and reliable measure of gender variance. It provides clinicians, researchers, and educators with a culturally appropriate tool to assess gender expression and support inclusive practices in both community and clinical contexts.

## 1. Introduction

Gender identity is increasingly understood as a multifaceted and fluid construct, shaped by psychological, social, and cultural processes [1]. Contemporary perspectives have moved beyond binary classifications, recognizing that individuals may experience and express their gender in ways that diverge from traditional norms associated with their assigned sex at birth [2,3]. Within this broader framework, gender variance refers to the degree to which a person’s self-perceived gendered traits—such as masculinity and femininity—differ from societal expectations [4,5]. This construct is particularly relevant in psychological and health research, as it intersects with experiences of stigma, visibility, and social acceptance, and may have implications for mental and physical well-being [6,7,8].

Despite growing international efforts to develop inclusive research tools, many psychometric instruments for assessing gender diversity remain unavailable in non-English-speaking contexts [9]. This limitation hinders culturally sensitive research and clinical practice, particularly in countries like Italy where gender diversity has received comparatively less empirical attention [10]. The Gender Variance Scale (GVS) was originally developed to provide a quantitative, self-report measure of gender variance, accounting for both self-perceived masculinity and femininity across behavioral and identity domains [11]. The scale was designed to be independent of gender identity categories, allowing individuals to describe their own experience of gendered traits without requiring alignment with binary norms. Initial validation studies demonstrated good psychometric properties in U.S.-based samples, supporting its potential as a flexible and inclusive tool for measuring gender diversity. However, the validity and applicability of the GVS in other cultural contexts remain unexplored. Adapting and validating the scale in the Italian language is a necessary step toward expanding the international use of this instrument and capturing culturally situated expressions of gender variance. Such validation efforts are particularly important in countries where public discourse around gender nonconformity is still evolving and where measurement tools may be limited to binary or pathologizing frameworks [12].

In addition to evaluating the scale’s internal structure and reliability, assessing its external validity is essential. One approach is to examine how gender variance relates to individuals’ perceptions of their overall health and functioning. Although not a direct measure of quality of life, the SF-12 provides a reliable assessment of self-perceived physical and mental health and is frequently used in population studies as a proxy for broader health-related outcomes [13,14]. Given that gender variance is often associated with minority stress, discrimination, and lack of access to affirming care, it is plausible to expect a relationship between higher levels of gender variance and lower perceived health, especially in environments where gender diversity is not fully supported [7,15].

The present study aims to validate the Italian version of the GVS in a mixed sample of participants from both the community and a clinical setting. Although several instruments for assessing gender variance are available in English and other languages, no validated tool currently exists in Italian. This adaptation is particularly relevant, as research and clinical practice in Italy still lack culturally appropriate and psychometrically sound measures for assessing gender identity and variance. Given that the original version of the GVS demonstrated a two-factor structure reflecting masculinity and femininity dimensions, we employed confirmatory factor analysis (CFA) to test whether this structure is replicated in the Italian context. In addition to examining the factorial structure, we evaluated the scale’s internal consistency and assessed its convergent validity by exploring correlations with the physical and mental health components of the SF-12, a widely used measure of self-perceived health. To further investigate the scale’s construct validity, we examined whether GVS scores differentiate between individuals identifying as binary and non-binary, as gender diversity is expected to vary along this dimension. By providing a culturally and psychometrically robust tool for assessing gender variance in Italian-speaking populations, this study contributes to the advancement of inclusive psychological research and practice.

## 2. Materials and Methods

### 2.1. Participants

A total of 356 individuals participated in this study. Participants were recruited from two sources: (1) the clinical sample consisted of TGD individuals enrolled at a specialized gender clinic at the University-Hospital of Padua (Italy), and (2) the comparison sample of cisgender individuals was drawn from the general population through an open call for psychological testing. Participation was voluntary and anonymous. Inclusion criteria for both groups included being 18 years of age or older, fluency in Italian, and provision of informed consent. Participants presenting with significant cognitive impairments or acute-phase psychiatric conditions likely to interfere with the reliability of self-reported information (e.g., psychotic or manic episodes) were excluded from the study.

The study was approved by the local ethics committee. All participants provided written informed consent prior to participation, in accordance with the Declaration of Helsinki. Data collection was carried out through anonymous self-report questionnaires.

### 2.2. Materials

The GVS consists of ten items, each rated on a 9-point Likert scale (1 = “not at all” to 9 = “completely”), assessing self-perceived traits related to masculinity and femininity [11]. Five items measure masculinity (e.g., feeling, acting, or looking masculine) and five items assess femininity (e.g., feeling, acting, or looking feminine), resulting in two subscale scores ranging from 5 to 45. In line with the original scoring system [11], a composite gender variance score was calculated to capture deviation from traditional gender norms, adjusted for sex assigned at birth. Specifically, for participants assigned male at birth, the formula was: (45–masculinity score) + (femininity score–5); for those assigned female at birth, it was: (45–femininity score) + (masculinity score–5). This computation yields a total GVS score ranging from 0 to 80, with higher values indicating greater gender variance, regardless of current gender identity. The scale was scored so that a cisgender individual with stereotypically gender-conforming responses would receive a lower score, whereas individuals who describe themselves with more mixed or non-normative gender traits receive higher scores.

The SF-12 [14] was used to assess perceived physical and mental health functioning. The SF-12 yields two composite scores: the Physical Component Summary (PCS) and the Mental Component Summary (MCS). Higher scores indicate better self-perceived health status. The Italian version of the SF-12 has been validated and widely used in health research [16,17]. In this sample, internal consistency was acceptable to good, with Cronbach’s α = 0.78 for the PCS and α = 0.83 for the MCS.

In addition to the study questionnaires, participants completed a brief demographic survey, which included items on age, weight, height, sex assigned at birth, and current gender identity. Participants were asked to self-identify their gender as either binary (man/woman) or non-binary (e.g., non-binary, genderqueer, fluid, or other identities not strictly aligned with binary categories). This variable was used to test the scale’s capacity to differentiate between binary and non-binary individuals in terms of gender variance scores.

### 2.3. Translation Procedure

The GVS was translated into Italian following established international guidelines for cross-cultural adaptation of self-report measures. The procedure included: (1) forward translation by two independent bilingual researchers (PM, ET), (2) reconciliation of the two versions into a single consensus translation, (3) back-translation by a native English speaker blind to the original version (DDB), and (4) comparison and harmonization with the original English version to ensure semantic and conceptual equivalence. The back-translation was performed independently by two bilingual translators. Any discrepancies were discussed in consensus meetings with the research team until agreement was reached, ensuring conceptual and semantic equivalence with the original version. A pilot test with 10 individuals (cisgender and TGD) was conducted. Participants completed the 10-item Italian GVS and were asked to provide open-ended feedback regarding clarity, wording, and cultural relevance. No additional quantitative indicators were collected, as the pilot was intended to qualitatively evaluate the adequacy of the translation prior to the main study. Minor linguistic adjustments were made following participant feedback.

The finalized Italian version of the scale is presented in Table 1.

### 2.4. Statistical Analysis

All statistical analyses were performed using SPSS (v.25) and JASP (v.0.18). Descriptive statistics, including means, standard deviations, skewness, and kurtosis, were calculated for each GVS item to examine data distribution and variability. Internal consistency of the total scale and its subscales (masculinity and femininity) was evaluated using Cronbach’s alpha. Floor and ceiling effects were evaluated by examining the proportion of participants achieving the lowest or highest possible score, with effects considered present if more than 15% of participants fell into either category.

To verify the factor structure, a CFA was conducted in JASP to test the hypothesized two-factor model (masculinity and femininity), following the structure proposed [11]. Model fit was assessed using the Comparative Fit Index (CFI), Tucker–Lewis Index (TLI), Root Mean Square Error of Approximation (RMSEA), and Standardized Root Mean Square Residual (SRMR), adopting conventional cutoffs for acceptable and good fit (CFI/TLI ≥ 0.90, RMSEA ≤ 0.08, SRMR ≤ 0.08).

Convergent validity was examined by calculating Spearman’s correlations between GVS scores (subscales and total score) and the physical (PCS) and mental (MCS) health components of the SF-12. Group differences were analyzed using non-parametric tests. Specifically, Mann–Whitney U tests were used for two-group comparisons (e.g., binary vs. non-binary; cisgender vs. TGD), while Kruskal–Wallis H tests with Bonferroni-corrected Mann–Whitney U post hoc tests were applied to evaluate differences across multiple gender identity categories (cisgender men, cisgender women, transgender men, transgender women, and non-binary individuals). Finally, a subsample of participants, including both cisgender and TGD individuals (*n* = 63), completed the GVS a second time after an interval of more than one month (mean interval = 31.00 days, SD = 1.2; range = 29–33) to assess test–retest reliability. Temporal stability was evaluated using both Pearson correlation coefficients and intraclass correlation coefficients (ICC), computed via a two-way mixed-effects model, absolute agreement, with 95% confidence intervals.

## 3. Results

The final sample comprised 356 participants: 192 individuals who identified as TGD and 164 cisgender individuals. Shapiro–Wilk tests indicated non-normal distributions for age, BMI, and education across groups. Therefore, Mann–Whitney U tests were conducted, which confirmed no significant differences between TGD and cisgender participants in age (U = 15,707, *p* = 0.95), education (U = 14,211, *p* = 0.13), or BMI (U = 17,030, *p* = 0.15). These results indicate that the two groups were broadly comparable in sociodemographic characteristics. All ten items of the GVS were completed by all participants, with no missing data. Item means ranged from 4.19 to 5.22, and standard deviations from 2.39 to 3.40, indicating acceptable variability. Skewness and kurtosis values for all items were within ±1.5, suggesting near-normal distributions. No floor or ceiling effects were observed.

Internal consistency was high across both subscales and the total scale. The femininity subscale had a Cronbach’s α of 0.86, the masculinity subscale α = 0.84, and the total scale α = 0.89.

### 3.1. Confirmatory Factor Analysis

**Figure 1:**
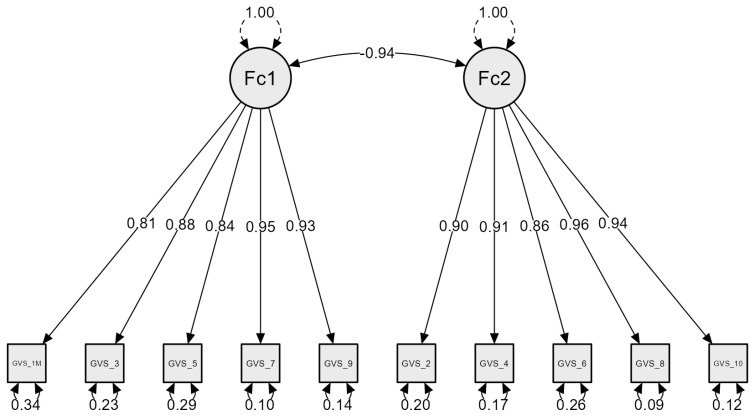
Confirmatory factor analysis of the Italian version of the Gender Variance Scale (GVS). Standardized factor loadings are shown on the paths from latent variables (Fc1: Masculinity, Fc2: Femininity) to observed items. Residual variances are displayed beneath each item. The correlation between latent factors is indicated by the curved arrow.

To evaluate the factorial structure of the GVS, a CFA was conducted on the 10-item scale using the original two-factor model proposed [11], with five items loading on the femininity factor and five on the masculinity factor. The model demonstrated adequate fit to the data: χ^2^(34) = 73.165, *p* < 0.001; Comparative Fit Index (CFI) = 0.916; Tucker–Lewis Index (TLI) = 0.905; Root Mean Square Error of Approximation (RMSEA) = 0.078; Standardized Root Mean Square Residual (SRMR) = 0.074. All factor loadings were statistically significant (*p* < 0.001). The correlation between the two latent factors (masculinity and femininity) was strong and negative (r = −0.94, *p* < 0.001), indicating that higher self-perceived masculinity was associated with lower self-perceived femininity. See Figure 1 for details.

### 3.2. Convergent Validity with Health-Related Quality of Life

TGD individuals reported significantly lower mental health scores than cisgender participants (MCS: TGD M = 47.99, SD = 8.43; cisgender M = 51.29, SD = 8.29; Mann–Whitney U = 12,254.5, *p* < 0.001, r = 0.19). For physical health, however, no significant difference emerged between groups (PCS: TGD M = 48.76, SD = 8.93; cisgender M = 49.05, SD = 8.19; Mann–Whitney U = 15,322.5, *p* = 0.736, r = 0.02). These findings support the scale’s convergent validity in reflecting minority stress-related disparities, particularly in mental health.

### 3.3. Group Differences in Gender Variance and Health Outcomes

TGD individuals had significantly higher GVS total scores than cisgender participants (M = 20.02, SD = 11.58 vs. M = 13.79, SD = 10.39; U = 20,664, *p* < 0.001, r = 0.32). When restricting the sample to binary participants only, the difference remained significant (M = 21.87, SD = 10.99 vs. M = 13.79, SD = 10.39; U = 19,362.5, *p* < 0.001, r = 0.42). No significant differences emerged for masculinity (M = 24.79, SD = 13.01 vs. M = 25.87, SD = 13.99; U = 14,913, *p* = 0.445, r = −0.05) or femininity (M = 20.86, SD = 12.56 vs. M = 23.48, SD = 14.49; U = 14,209, *p* = 0.135, r = −0.09). Significant differences emerged across gender identity groups on all three GVS subscales: masculinity, femininity, and overall gender variance (see Table 2). Cisgender men and trans men reported the highest masculinity scores, whereas cisgender and trans women showed higher femininity scores. Non-binary participants exhibited intermediate levels of both masculinity and femininity, reflecting a more androgynous profile. Gender variance scores were significantly higher in trans and non-binary individuals compared to cisgender participants, with non-binary individuals showing the highest overall variance. Post hoc analyses revealed significant pairwise differences across most group comparisons, particularly between cis and TGD individuals on each subscale.

Non-binary individuals (*n* = 25) reported poorer health outcomes than binary individuals (*n* = 331). Differences in PCS did not reach significance (M = 46.15, SD = 7.63 vs. M = 50.56, SD = 9.46; U = 3.23, *p* = 0.069, r = −0.22). In contrast, MCS scores were substantially lower among non-binary participants (M = 38.80, SD = 3.33 vs. M = 51.32, SD = 8.97; U = 782.5, *p* < 0.001, r = −0.81). The composite GVS score was also significantly higher among non-binary compared to binary individuals (M = 17.87, SD = 11.43 vs. M = 7.64, SD = 6.89; U = 1965.5, *p* < 0.001, r = −0.52).

### 3.4. Influence of Education and BMI on GVS Scores

Spearman correlations showed that BMI was modestly correlated with masculinity (ρ = 0.13, *p* = 0.020) and negatively with femininity (ρ = −0.16, *p* = 0.003). Education was negatively associated with the GVS total score (ρ = −0.17, *p* = 0.002), but not with the subscales. No robust evidence for significant interaction effects between education or BMI and group emerged in non-parametric analyses, although the correlations suggest that the association between BMI and gendered traits may be stronger in TGD participants.

### 3.5. Test–Retest Reliability

Test–retest reliability was examined in a subsample of participants who completed the GVS at two time points, spaced more than one month apart. Pearson correlation coefficients between baseline (T0) and follow-up (T1) scores indicated strong temporal stability. The masculinity subscale showed excellent reliability (r = 0.99, *p* < 0.001), the femininity subscale demonstrated good stability (r = 0.83, *p* < 0.001), and the overall GVS total score, reflecting composite gender variance, also showed high reliability across time (r = 0.87, *p* < 0.001).

To complement these findings, ICC were calculated using a two-way mixed-effects model. The GVS total score demonstrated excellent agreement, with an ICC of 0.996 (95% CI [0.993, 0.997], *p* < 0.001) for average measures and 0.992 (95% CI [0.987, 0.995], *p* < 0.001) for single measures. Subscale analyses confirmed high reliability: the masculinity subscale had an ICC of 0.945 (95% CI [0.911, 0.966], *p* < 0.001), and the femininity subscale an ICC of 0.948 (95% CI [0.917, 0.968], *p* < 0.001). These results support the strong test–retest reliability and temporal consistency of the Italian version of the GVS.

## 4. Discussion

The present study provides strong support for the Italian adaptation of the GVS as a psychometrically sound instrument for assessing self-perceived gender expression across diverse populations. The results align with contemporary models that conceptualize gender as a dimensional and context-sensitive construct, rather than a fixed binary category [1,18]. By preserving the original two-factor structure (masculinity and femininity), the Italian version of the GVS captures the spectrum of gendered self-perceptions while remaining inclusive of both cisgender and TGD individuals.

Notably, the scale demonstrated differential sensitivity across gender identity groups. The lower total GVS scores observed in non-binary participants—despite being counterintuitive at first glance—may reflect a meaningful departure from binary gender constructs. Rather than indicating reduced gender variance, these scores may represent non-identification with traditional masculine and feminine traits, aligning with conceptualizations of non-binariness as a distinct positionality rather than a midpoint on a continuum [11,12]. In the Italian sociocultural context, where non-binary identities remain less visible and institutionally recognized than binary transgender identities, such patterns may also reflect cultural influences on self-expression. Moreover, because the GVS is structured around two binary dimensions, its sensitivity to androgynous or fluid identity profiles may be limited, underscoring the need for future refinements to better capture non-binary experiences. Overall, the GVS—particularly its total score—may serve as a marker of divergence from binary gender alignment, though future work should test adaptations that enhance sensitivity to diverse non-binary trajectories. The associations between GVS scores and perceived physical and mental health are consistent with minority stress theory [19], which posits that gender nonconformity may expose individuals—especially TGD people—to increased psychosocial stress, discrimination, and reduced access to affirming care. These dynamics can in turn influence well-being, suggesting that measures of gender variance may have clinical utility in identifying at-risk individuals or tailoring interventions [10,15]. Furthermore, the influence of education and BMI on GVS scores invites consideration of how gender expression is shaped by intersecting sociocultural and embodied factors. For example, cultural ideals linking body shape to gender norms may influence how individuals perceive and report their gendered traits [20,21,22], particularly within TGD populations navigating body image and gender congruence. Previous studies have shown that transgender men have a significantly higher BMI than cisgender women, a finding consistent with our observed correlation between BMI and masculinity scores [23].

### 4.1. Clinical and Research Implications

The Italian GVS offers clinicians and researchers a valid tool to assess gender variance without prescriptive or pathologizing assumptions. It enables a more nuanced understanding of gender identity expression in both clinical assessments and psychosocial studies. Given its demonstrated associations with mental and physical health, the GVS may inform psychological formulation, care planning, and risk assessment, particularly in settings working with gender-diverse populations. Its applicability to both binary and non-binary individuals makes it especially relevant for inclusive clinical practice and gender-affirming interventions.

In research contexts, the scale provides a dimensional metric that transcends traditional diagnostic or identity-based categories, enabling investigations into the role of gender variance in psychopathology, well-being, and social adaptation. Importantly, the instrument supports person-centered and culturally sensitive methodologies, particularly in Italian and other non-English-speaking populations where validated tools are limited. Beyond clinical and research settings, the GVS may also be useful for educators seeking to promote awareness and inclusivity in schools and universities, and for policymakers aiming to capture population-level data on gender variance to inform gender-sensitive health and social services.

### 4.2. Limitations and Future Directions

Despite the strengths of the current study, several limitations warrant attention. First, the cross-sectional design precludes causal inference regarding associations between gender variance and health outcomes. Second, while the sample was diverse in terms of gender identity, it may not capture the full range of socio-demographic and regional variability present within the Italian population. In particular, the distribution of age and education levels was not balanced, and most participants were recruited from specific regions, which may limit the generalizability of findings to the broader Italian context. Third, although the scale showed good test–retest reliability, the relatively short retest interval (29–33 days) limits conclusions about long-term stability. Finally, the TGD participants were recruited from a specialized clinical center, which may introduce selection bias and limit the generalizability of findings to the broader TGD community.

The scoring algorithm, originally developed based on sex assigned at birth, may not fully account for non-binary forms of gender expression. Future studies could explore alternative scoring approaches (e.g., distance-based metrics or multidimensional scaling) that better reflect the complexity of non-binary experiences. Moreover, the incorporation of qualitative data could help refine item content and interpretation, ensuring the tool remains inclusive and sensitive across cultural contexts.

## 5. Conclusions

The Italian version of the GVS demonstrates strong psychometric properties and validity across a diverse sample of cisgender and TGD individuals. Its ability to capture nuanced patterns of gender expression—including meaningful differences between binary and non-binary identities—supports its use as a flexible and inclusive measure in both research and clinical practice. By bridging the gap in gender variance assessment tools for Italian-speaking contexts, the GVS contributes to the advancement of gender-affirming care and inclusive psychological science.

## Figures and Tables

**Table 1:** Gender Variance Scale (GVS): Original Items and Italian Translation.

	Original (English)	Italian Translation
	Many people describe themselves and others as some combination of feminine (girlish) and masculine (boyish) because of how we feel, act, talk or dress. The next questions are about how you describe yourself.	Molte persone si descrivono, o descrivono gli altri, come una combinazione di femminile e maschile in base a come si sentono, si comportano, parlano o si vestono. Le prossime domande si riferiscono al modo in cui descrivi te stesso/a.
1	On a scale of 1–9, where 1 means ‘not at all’, and 9 means ‘completely’, to what extent would you say that your interests are mostly those typical of a boy/young man/masculine person?	Su una scala da 1 a 9, dove 1 significa “per niente” e 9 significa “completamente”, in che misura i tuoi interessi sono tipicamente da maschio?
2	On a scale of 1–9, where 1 means ‘not at all’, and 9 means ‘completely’, to what extent would you say that your interests are mostly those typical of a girl/young woman/feminine person?	Su una scala da 1 a 9, dove 1 significa “per niente” e 9 significa “completamente”, in che misura i tuoi interessi sono tipicamente da femmina?
3	On a scale of 1–9, where 1 means ‘not at all’, and 9 means ‘completely’, to what extent would you say that you do most things in a manner of a boy/young man/masculine person?	Su una scala da 1 a 9, dove 1 significa “per niente” e 9 significa “completamente”, in che misura il tuo modo di fare sia tipicamente maschile?
4	On a scale of 1–9, where 1 means ‘not at all’, and 9 means ‘completely’, to what extent would you say that you do most things in a manner of a girl/young woman/feminine person?	Su una scala da 1 a 9, dove 1 significa “per niente” e 9 significa “completamente”, in che misura il tuo modo di fare sia tipicamente femminile?
5	On a scale of 1–9, where 1 means ‘not at all’, and 9 means ‘completely’, how masculine do you think you look?	Su una scala da 1 a 9, dove 1 significa “per niente” e 9 significa “completamente”, in che misura pensi di avere un aspetto mascolino?
6	On a scale of 1–9, where 1 means ‘not at all’, and 9 means ‘completely’, how feminine do you think you look?	Su una scala da 1 a 9, dove 1 significa “per niente” e 9 significa “completamente”, in che misura pensi di avere un aspetto femminile?
7	On a scale of 1–9, where 1 means ‘not at all’, and 9 means ‘completely’, how masculine do you feel?	Su una scala da 1 a 9, dove 1 significa “per niente” e 9 significa “completamente”, quanto ti senti maschile?
8	On a scale of 1–9, where 1 means ‘not at all’, and 9 means ‘completely’, how feminine do you feel?	Su una scala da 1 a 9, dove 1 significa “per niente” e 9 significa “completamente”, quanto ti senti femminile?
9	On a scale of 1–9, where 1 means ‘not at all’, and 9 means ‘completely’, how male do you feel?	Su una scala da 1 a 9, dove 1 significa “per niente” e 9 significa “completamente”, quanto ti senti uomo?
10	On a scale of 1–9, where 1 means ‘not at all’, and 9 means ‘completely’, how female do you feel?	Su una scala da 1 a 9, dove 1 significa “per niente” e 9 significa “completamente”, quanto ti senti donna?
11	What was your sex registered at birth (The sex put on your birth certificate?) Male/Female	Qual è il tuo sesso registrato alla nascita (cioè quello indicato sui documenti di nascita)? Maschio/Femmina

Note. All items are rated on a 9-point Likert scale (1 = “not at all”; 9 = “completely”). Italian items were adapted using a forward–backward translation method and culturally validated. The item on birth-assigned sex is used in scoring the total GVS index as per the original authors’ guidelines.

**Table 2:** Group Comparisons on GVS Subscales: Means, Standard Deviations, and ANOVA Results.

Subscale	Group	M	SD	H	*p*	Post Hoc
Masculinity	Cisgender men	38.80	4.63	247.49	<0.001	Trans men < Cis men (*p* < 0.001) Trans men < Cis women (*p* < 0.001) Non-binary < Cis men (*p* < 0.001) Trans women < Cis men (*p* < 0.001) Trans men < Trans women (*p* = 0.002) Non-binary < Cis women (*p* < 0.001) Trans women < Cis women (*p* < 0.001)
	Cisgender women	34.96	6.32			
	Transgender men	12.89	8.24			
	Transgender women	21.28	8.71			
	Non-binary	13.25	9.16			
Femininity	Cisgender men	9.85	5.60	238.48	<0.001	Trans women < Cis women (*p* < 0.001) Trans women < Trans men (*p* < 0.001) Trans women < Non-binary (*p* < 0.001) Cis men < all others (all *p* < 0.001) Non-binary < Cis women (*p* < 0.001) Non-binary < Trans men (*p* = 0.007)
	Cisgender women	36.78	5.13			
	Transgender men	33.10	7.04			
	Transgender women	12.17	6.77			
	Non-binary	17.80	10.02			
GVS Total	Cisgender men	11.05	5.19	71.64	<0.001	Trans women > Cis men (*p* < 0.001) Trans men > Cis men (*p* < 0.001) Cis women > Cis men (*p* = 0.004) Non-binary < Cis women (*p* < 0.001) Non-binary < Trans women (*p* < 0.001) Non-binary < Trans men (*p* < 0.001)
	Cisgender women	16.47	9.54			
	Transgender men	20.21	10.68			
	Transgender women	23.17	10.59			
	Non-binary	7.64	5.73			

Note. Means and standard deviations are reported for descriptive purposes. Group differences were tested using Kruskal–Wallis H tests with pairwise Mann–Whitney U tests (Bonferroni correction) for post hoc comparisons.3.4. Binary vs. Non-Binary Participants.

## Data Availability

The data presented in this study are available on reasonable request from the corresponding author. Restrictions apply due to ethical considerations and approval by the Institutional Ethics Committee, given the sensitivity of the transgender and gender-diverse population under investigation.
